# Inflammatory Myofibroblastic Tumor Invading the Left Atrium: Report of a Rare Case

**Published:** 2018-01

**Authors:** Hikmet Sahratov, Adem Guler, Mustafa Kurkluoglu, Bahadır Calişkan, Fahri Gurkan Yesil, Murat Tavlasoglu, Mehmet Ali Sahin

**Affiliations:** 1 *Department of Cardiovascular Surgery, Gulhane Military Medical School, Ankara, Turkey.*; 2 *Department of Cardiovascular Surgery, Etimesgut Military Medical Hospital, Ankara, Turkey.*

**Keywords:** *Lung neoplasms*, *Heart atria*, *Child*

## Abstract

Inflammatory myofibroblastic tumors (IMTs) of the lung are rare solid tumors and usually affect children and young adults. We describe an unusual form of an IMT of the left lower lobe invading the left atrium. A 9-year-old male patient with recurrent cough was referred for an evaluation of left-lung pneumonia. Transthoracic needle biopsy was performed, and the histopathological examination showed mixed inflammatory cells. Accordingly, an IMT was considered. Left lower lobectomy was performed. A portion of the tumor invading the left atrium was resected together with the intact atrial wall. The postoperative period was uneventful, and the patient was discharged on the sixth postoperative day.

## Introduction

Inflammatory myofibroblastic tumors (IMTs) are rare tumors, but they can involve various organs such as the liver, gastrointestinal tract, mediastinum, and retroperitoneum.^1^ IMTs most often affect the lungs of children under the age of 16 and young adults. We describe an unusual form of an IMT of the left lower lobe with invasion of the left atrium.

## Case Report

**Figure 1:**
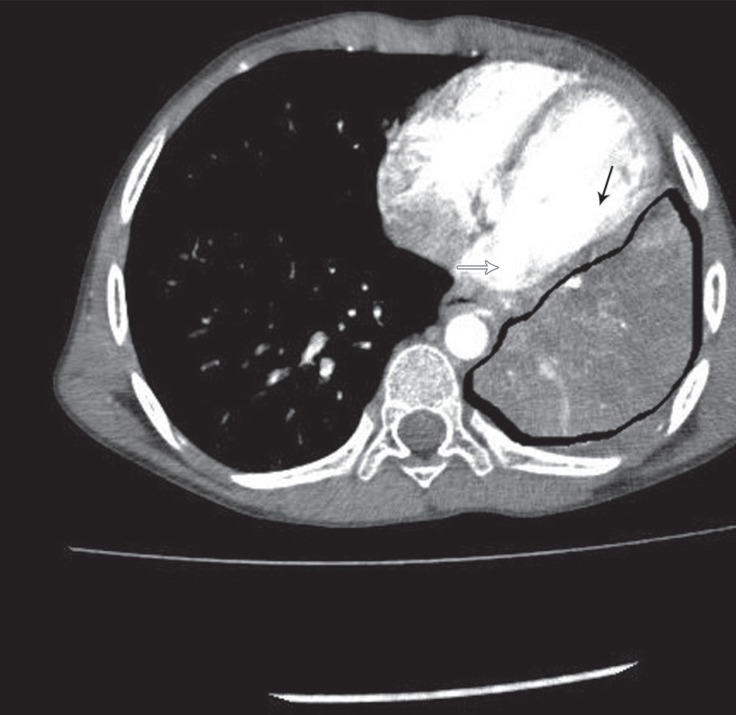
Computed tomography image of the inflammatory myofibroblastic tumor, invading the left atrium. White thick arrow: left atrial wall invaded by the tumor; black thin line: left ventricular wall; area surrounded by the black thick line: inflammatory myofibroblastic tumor with the lower lobe

A 9-year-old male patient with recurrent cough was referred for an evaluation of left-lung pneumonia. The patient’s medical history revealed that he had been treated for this complaint several times. The chest X-ray showed an increased opacity in the left lower lobe. He was given amoxicillin/clavulanic acid, inhaled prednisolone, and salbutamol, which conferred only a partial reduction in symptoms. The chest computed tomography illustrated a mass of parenchymal origin in the lower lobe of the lung invading the left atrium (Figure 1). Transthoracic needle biopsy was performed, and the histopathological examination demonstrated mixed inflammatory cells. An IMT was, therefore, considered.

The operative approach was considered for the patient to manage the invasive tumor. A left thoracotomy incision revealed the tumor invading the left atrium. Subsequently, left lower lobectomy was performed. Next, a portion of the tumor invading the left atrium was resected together with the intact atrial wall. Finally, the left atrium was repaired with pledgeted sutures. The procedure did not require cardiopulmonary bypass. After the operation was terminated, the histopathological examination of the mass confirmed the diagnosis of an IMT. The postoperative period was uneventful, and the patient was discharged on the sixth postoperative day.

## Discussion

IMTs have been known by different names such as inflammatory plasma cell granulomas, pseudotumors, fibroxanthomas, xanthofibromas, and xanthomas. Their etiology is, however, still controversial. Some researchers claim that they are true neoplasms, whereas others believe that they are non-neoplastic processes like metabolic disturbances, viral origins, or antigen-antibody interactions with an unknown agent.^1^ Although histopathologically benign, IMTs may be indistinguishable from malignancies because of their local invasive nature and the possibility of recurrence.^2^ In our patient, we detected an invasion of the left atrium by the tumor originating from the left lung. 

IMTs often occur in the lung, followed by the small and large intestines, mesentery, mediastinum, retroperitoneum, omentum, and diaphragm. Less common sites are the thyroid, brain, spinal cord meninges, and liver.

The clinical presentation depends on the organ from which the tumor originates. IMTs of the lung are usually asymptomatic; nevertheless, some patients present with cough, shortness of breath, chest pain, dysphagia, and hemoptysis.^1^ The symptoms of the IMTs of the lung are often related to the location of the lesion, whether it is parenchymal, endobronchial, or mediastinal. Kim et al. reported a series of 28 patients in whom the locations of the lesions were parenchymal (85.7%), endobronchial (10.7%), and endotracheal (3.6%).^3^

IMTs are usually detected by routine chest X-rays. Laboratory evidence is nonspecific and may show hypochromic microcytic anemia, increased immunoglobulins, and elevated erythrocyte sedimentation rate. A physical examination yields less information. The computed tomography findings of IMTs usually manifest like well-circumscribed, solitary peripheral pulmonary nodules.^2^ The definitive diagnosis of IMTs is made by transbronchial biopsy or transthoracic needle biopsy, yielding histopathologic findings of spindle-shaped cells with a chronic inflammatory component consisting of plasma cells, lymphocytes, and occasional histiocytes.^2^

The best treatment option is complete surgical resection of the tumor.^2^ Obtaining a tumor-negative margin is important to determine the extent of resection. Cerfolio et al.^4^ reported a 60%recurrence rate in patients who had received incomplete resection.^4^ In patients whose IMTs are deemed unresectable or in patients who are medically inoperable, there are reports of success with treatment employing corticosteroids, radiotherapy, and chemotherapy.^5^

## Conclusion

Inflammatory myofibroblastic tumors (IMTs) are rare benign neoplasms that are clinically and radiologically similar to cancer. The left atrial invasion is rare. The complete resection of the mass is the best treatment option. Surgery is both diagnostic and therapeutic. Recurrent cases may need re-resection. In severe cases, cardiac invasion may require the employment of cardiopulmonary bypass. It is, therefore, advisable that a heart-lung machine be available. In addition, perfusionists and appropriate supplies for cardiopulmonary bypass should be considered in planning the surgical procedure. 

## Notes:

***This paper should be cited as:*** Sahratov H, Guler A, Kurkluoglu M, Calişkan B, Yesil FG, Tavlasoglu M, Sahin MA. Inflammatory Myofibroblastic Tumor Invading the Left Atrium: Report of a Rare Case. J Teh Univ Heart Ctr 2018;13(1)24-26.
